# Influence of Processing Parameters on Tensile Properties of SLS Polymer Product

**DOI:** 10.3390/polym10111208

**Published:** 2018-10-31

**Authors:** Ana Pilipović, Tomaž Brajlih, Igor Drstvenšek

**Affiliations:** 1 Faculty of Mechanical Engineering and Naval Architecture, University of Zagreb, Ivana Lucica 5, 10000 Zagreb, Croatia; 2 Faculty of Mechanical Engineering, University of Maribor, Smetanova ulica 17, 2000 Maribor, Slovenia; tomaz.brajlih@um.si (T.B.); igor.drstvensek@um.si (I.D.)

**Keywords:** additive manufacturing, beam overlay ratio, equation, layer thickness, polymer, SLS, tensile properties

## Abstract

Polymer products manufactured by additive processes are today increasingly flooding the market. Given that they have broad application ranging from various consumer products to medicine and automotive industry, the products must satisfy certain mechanical properties. In the past studies of selective laser sintering (SLS) for polymer materials, the processing parameter of energy density has been confirmed which affects the tensile properties. Energy density depends on the laser beam speed, laser power and hatch distance; however, in this paper the existing mathematical model has been expanded by the overlay ratio and tests have been conducted how on the basis of the new mathematical model a product with good tensile properties (tensile strength, tensile strength at break, tensile modulus, tensile strain at break) can be manufactured. However, in parameter selection as well, the layer thickness and the manufacturing strategy also play a role, and they may shorten the time and reduce the cost necessary to manufacture a new product from the initial concept to production. The paper also provides a proposal of processing parameters (laser beam speed, laser power and energy density) depending on the manufacturing strategy and layer thickness.

## 1. Introduction

With additive manufacturing it is possible to produce parts with complex geometry [1]. To select the right additive technology, it is necessary to consider the number and volume of products, material and its properties, time of production, and cost [2]. The number of available materials is limited to some polymers (semicrystalline or crystalline), metals and ceramic [1,3]. This paper is focused on only one technology in additive manufacturing, selective laser sintering (SLS). SLS is a process used to produce parts from powdered materials using one or more lasers to selectively fuse or melt the particles at the surface, layer upon layer, in an enclosed chamber [1].

In SLS the material properties depend also on processing parameters: scan strategy, laser power, laser beam speed, spot diameter of the laser beam, temperature of working chamber, material shrinkage, beam offset, layer thickness, hatch distance, powder properties and also on accuracy of STL model, cutting into layers, machine resolution, etc. [4,5,6,7,8,9].

The sintering process and properties of products (surface roughness, dimensional accuracy, tensile properties, flexural properties, and time of manufacturing) are affected by laser processing parameters. Processing parameters can be changed separately for the external layers (contour of the object) and product interior layers (hatching). The mechanical properties as well as better sintering of particles are affected by energy density on the external layers and the interior of the product. According to the available literature and experiments done by other authors, the processing parameters are calculated through the energy density. Although all of them for calculation of energy density use laser power and speed, some of the authors state that energy density depends on hatch distance while other in their calculation use laser beam diameter into consideration [5,10,11,12,13,14,15]:

(1) $$ED=\frac{P}{v\cdot h}\;$$

(2) $$ED=\frac{P}{v\cdot d}\;$$

where is:

*ED*  energy density, J/mm^2^
*P*  laser power, W
*v*  laser beam speed, mm/s
*h*  scan spacing, hatch distance, mm
*d*  laser beam diameter, mm

The value of the laser power during production depends on the type of material, e.g., polymers or metals, and the layer thickness. Laser beam speed and laser power during the production of the contour are lower in relation to the laser speed and power during the production of the interior hatching of individual layer. Energy density and the production time change with the change in laser speed [12,13,14,15,16]. In the experiments done by authors Caulfield et al. only scan spacing and energy density lower than 0.028 J/mm^2^ were used as parameters. They concluded that tensile properties are better if energy density is higher, but maximum energy input to the product without decreasing the tensile properties in not known. Also from this paper cannot be concluded how to set the parameters within energy density to obtain the proposed value of 0.028 J/mm^2^ which gave the highest value of the tensile properties [11].

The experiment analyzed by Analysis of variance (ANOVA) with the above-mentioned Equation (1) was carried out by authors Singh et al. They conducted the experiment on how processing parameters calculated with equation for *ED* influence the density and hardness of a product built with polyamide. As a consequence of using Equation (1) for *ED*, in their results a significant deviation from lack of fit is shown (deviation from the model is very large, i.e., it is only 1.8% that the corresponding analysis follows the model/equation). Also, R-squared is only 0.46 which is very low [5]. In another work by the same author Singh et al. it was concluded that the scan spacing is the most significant parameter in Equation (1), but also there were a significant deviation in the lack of fit [6,17].

From given can be concluded that it is necessary to check if the equation for building products in SLS needs to be extended with some new or additional parameters which is the focus of this paper.

## 2. SLS

**Figure 1:**
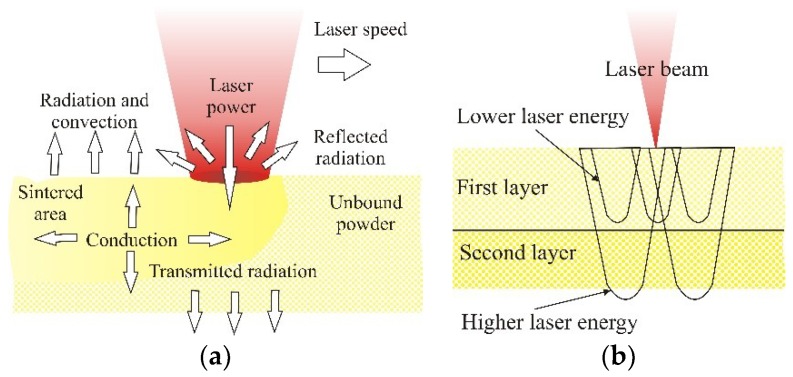
(**a**) Laser power influence on the sintering process, (**b**) selection of laser power/energy input [19,20].

SLS is based on selectively fusing powder to shape an object. The process setup mainly consists of a laser as an energy source, a mirror system, a powder bed which is mounted on a movable platform and a levelling roller. To create an object, the levelling roller moves a thin film of powder onto the movable platform, where the laser beam, guided by the mirror system, melts the selected areas of the powder bed. When heated sufficiently, the particles fuse together and form a layer. After cooling, the platform is lowered, and the process starts again. By successively adding layers of varying shapes and joining them to previous layers in the melting process, a solid body is generated. The energy demand for creating an object depends on a wide variety of materials and machinery-specific influencing parameters [18]. For good sintering of two layers there must be adequate laser energy (Figure 1a). At lower laser energy the layers will not fuse together, but if there is too much energy (Figure 1b) the product will deform (warping) and deviate from its dimensions.

After finishing all layers, the part is left to cool down to room temperature which affects dimensional accuracy, shrinkage of part and reduction of heat deformations [13,21].

## 3. New Equation for Determining Energy Density in SLS

### 3.1. The Laser Parameters in SLS

Laser parameters can affect the properties of the products (for example surface roughness and mechanical properties) and the sintering [13]. For the contour and for hatching, laser parameters can be adjusted separately.

**Laser Power***P* [W] is input as a percentage of the maximum laser source power. The input value depends on the type of the material and the layer thickness, with which the part is built. During building of a layer, the laser power for the contouring is generally smaller than that for the hatching distribution [13].

**Laser Scanning Speed***v* [mm/s] in production of the contour the laser speed is lower than in production of the hatching. With the change in the laser beam speed the energy in the material and the time of making the product are changed as well [13].

**Effective Diameter of the Laser Beam**—In SLS systems, the produced laser beam is focused down to a certain beam diameter where it contacts the powder surface. However, the diameter of the region where the particles are sintered (effective sintering range) is larger than the physical beam diameter. This range is denoted as the effective diameter of the laser beam, *D*_e_ [mm], which is proportional to the laser power and inversely proportional to the scanning speed of the laser. As the laser power and the laser speed settings for the contouring and hatching differ, the *D*_e_ during contouring (*D*_ec_) and hatching (*D*_eh_) also differs [13]. For easier representation, the effective and physical diameters are represented as circles, although their actual appearance depends on the *x* and *y* rotation of the scanning mirror.

**Figure 2:**
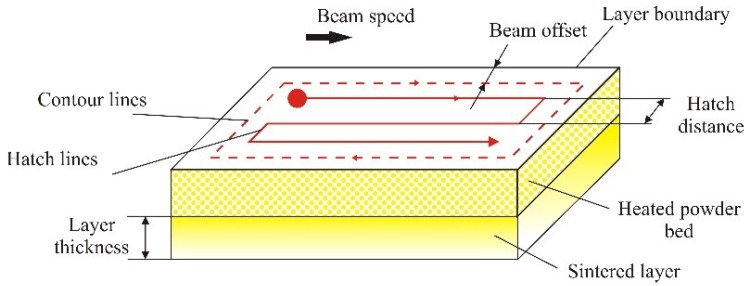
Hatch distance [22,23].

**Figure 3:**
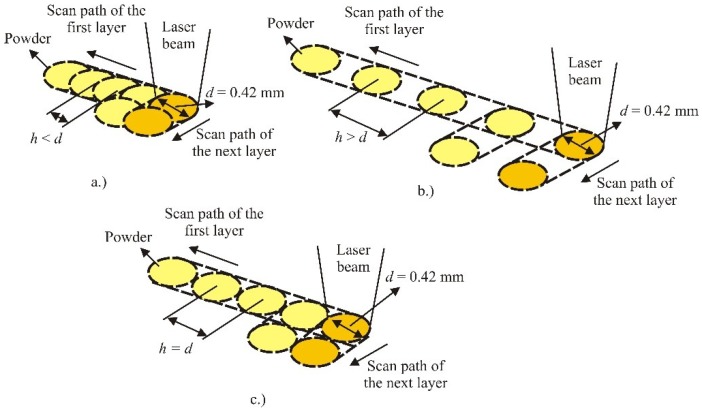
Scanning paths for sintering: (**a**) *h* < *d*, (**b**) *h* > *d* and (**c**) *h* = *d* [20].

**Hatch Distance**—The hatch distance *h* [mm] (Figure 2) must be smaller than the effective diameter of the laser beam during hatching, *D*_eh_, otherwise a connection between the hatching lines cannot be guaranteed (Figure 3) [13].

Equation $ED=\frac{P}{v\cdot h}$ comes from geometric proportions in Figure 2, so if hatch distance is larger or equal than the diameter of the laser beam (*h* ≥ *d*) then the equation is $ED=\frac{P}{v\cdot d}$. In all other cases, the first equation applies. These two equations do not include correlation of both parameters; hatch distance and laser beam diameter which are the basic principles of laser scanning the surface of powder. Scanning paths and explanation is shown in Figure 3. This is one more reason that these two equations must be modified.

Also, according to a contradictory opinion in the available literature with Equations (1) and (2) for energy density *ED*, which is one of the main parameters which affects mechanical properties, a pre-experiment for the tensile properties was done and it is concluded that the parameters depend on each other and that the equation must be pre-formulated and reconstructed [20].

From the principles of SLS, Figure 3, these two available equations and as outlined by previous papers laser beam diameter *d* and hatch distance *h* are essential parameters for mechanical properties and certainly both should be included in the calculation of energy density.

In the pre-experiment we expanded the equation with overlay ratio *x* = *d*/*h* [20]:

(3) $$\;ED=\frac{P}{h\cdot v}\cdot \frac{d}{h}\;$$

The experiment was conducted with machine *Formiga P100* and in this machine the laser beam diameter is *d* = 0.42 mm.

### 3.2. Strategy of Manufacturing Products in SLS

In all AM processes, the machine path traverses the internal cross section of the part. For example, the standard scanning strategy to produce parts using selective laser processes first scans the boundary contour and then moves inward to ”hatch” the internal cross section. Various process planning techniques have been developed to accommodate different AM technologies. Factors such as hatch spacing and degree of orientation are important parameters for determining the final mechanical properties of the part. In addition to these parameters, laser beam speed and layer thickness can have significant effects on part shrinkage and the final size of the part [22,24,25,26].

**Figure 4:**
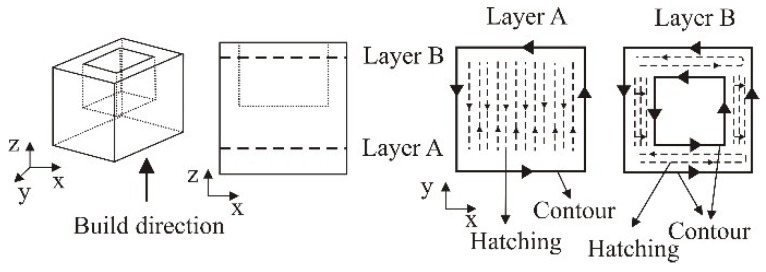
Strategy of manufacturing simple products [13].

The SLS equipment manufacturers offer various manufacturing strategies. The manufacturing strategies differ depending on the type of machine and manufacturer. The manufacturing strategy is a very important factor and its proper choice can significantly affect the properties of the manufactured product. Dimensional accuracy, surface quality, mechanical properties and time of product manufacturing are some of the reasons for the selection of different manufacturing strategies. Figure 4 shows the manufacturing strategy of a simple product [13].

In layer A the laser beam first makes the contour, and this is followed by making of the layer hatching. In scanning the hatching, the laser beam moves between the previously made contour, using one of the possible manufacturing strategies (in this example the movement is done along the simplest path of the laser beam). In layer B also first the contour is made, and the difference between layer A and layer B is that in layer B there is a contour also on the internal side. The complexity of layer B is at a higher level and the proper selection of the manufacturing strategy can have a significant influence [13].

In SLS, done in this paper with machine by manufacturer *EOS*, there are eight manufacturing strategies: contour and hatching, laser beam scanning direction, sorted, unsorted, skincore, mesh 2D, UpDownskin, edges [13,27].

**Figure 5:**
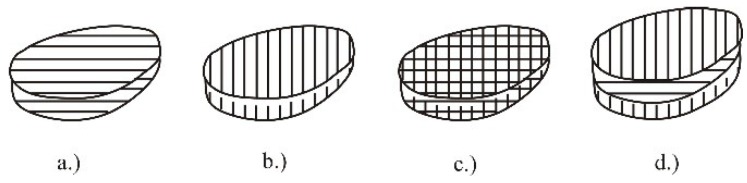
Scanning direction: (**a**) *x* axis, (**b**) *y* axis, (**c**) *xy* combination, (**d**) alternating [27].

For this experiment we used scanning direction alternating and sorted. Laser beam movement in layer sintering, (product hatching), can be performed in two directions, as presented in Figure 5a,b. Depending on the need and the desired properties it may be determined whether the layer sintering regarding the working platform, will move in direction *x* or *y*. The sintering of one layer can be done also in both directions (Figure 5c), but in alternating manner (Figure 5d), i.e., every second layer is sintered in the same direction. The selection of the sintering direction can have significant influence on the product properties [27].

**Figure 6:**
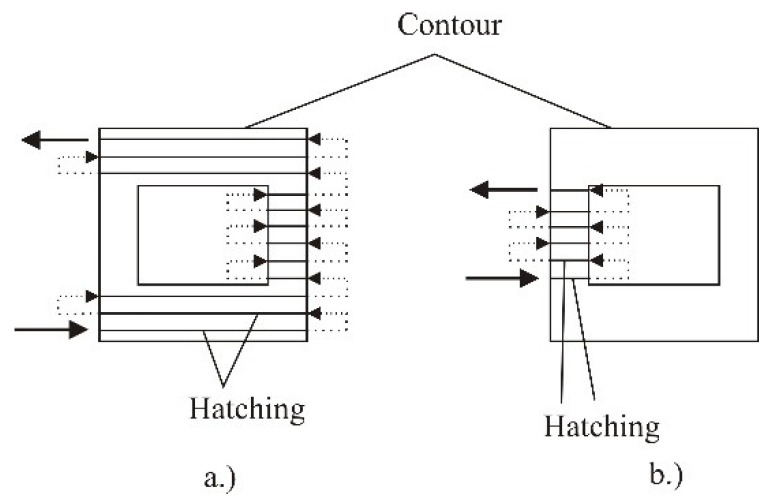
Manufacturing strategy sorted: (**a**) first phase, (**b**) second phase [27].

The manufacturing strategy *Sorted* (Figure 6), which will be applied in the experimental part of the paper, refers to the manufacturing of single layers of the product. The product layers are made in the shortest period, and the path is classified regarding the edge contours which significantly affects the manufacturing time. As presented in Figure 5 the layer manufacturing can be performed in several phases, depending on the product design. In making the second phase (Figure 6b) on the joints with the first phase voids or indents may occur which is at the same time a drawback of this manufacturing strategy. These drawbacks may significantly influence the product properties [27].

## 4. Experimental Part

The theory for the new equation has been shown on the tensile properties and it is described below.

All experiments in Section 4 were carried out with material polyamide PA 12 (material PA 2200 manufacturer *EOS* *GmbH Electro Optical Systems*, Krailling, Germany, with some constant processing parameters:

layer thickness = 0.1 mm;

chamber temperature = 172 °C;

beam offset = 0.15 mm;

material shrinkage along *x* axis 3.4%, along *y* axis 3.4%, along *z* axis at 0 mm 2.2% to *z* axis at 300 mm 1.6%;

alternating scanning direction;

manufacturing strategy sorted;

compensation of laser beam speed included.

Tensile properties are measured on universal testing machine *Messphysik Beta 50-5* (*Messphysik Materials testing GmbH,* Fürstenfeld, Austria), with a maximum loading force of 50 kN. The tensile testing was carried out at room temperature of 23 °C with speed *v* = 5 mm/min according to standard ISO 527:2012. Some properties of material PA2200 are [28]:

- average grain size 60 µm
- bulk density 0.435–0.445 g/cm^3^
- density of laser sintered part 0.9–0.95 g/cm^3^

**Figure 7:**
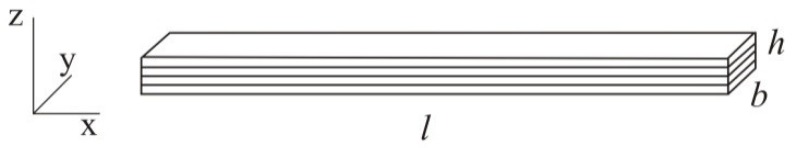
Orientation of the test specimen.

All test specimens are oriented as in Figure 7, and test specimens were placed in the lowest position of the working chamber. In one row, 9 test specimens can be placed, so that 3 test specimens of 3 experiment runs were placed in first row. After the first row there must be free space of only powder in the height of 5 mm, then the second row of 9 specimens, etc.

**Figure 8:**
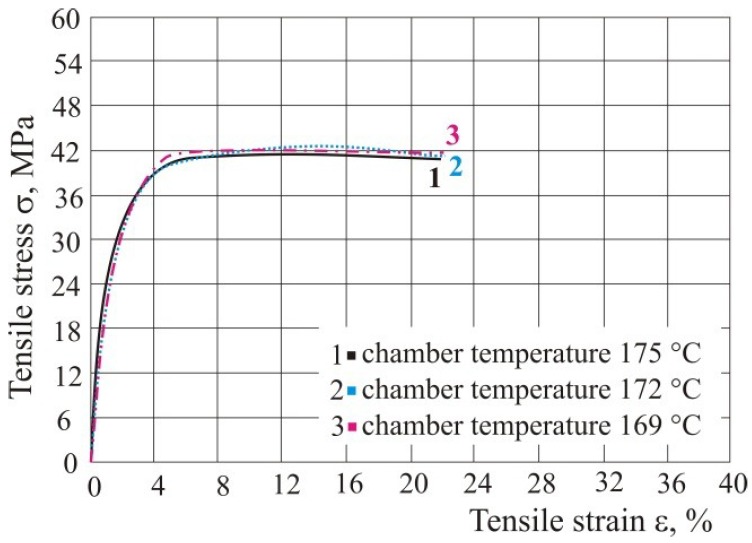
Influence of chamber temperature on the tensile properties.

The parameter that can be adjusted is also the working chamber temperature. The test was done with energy density of 0.05 J/mm^2^. When using polyamide, it is limited to the range of 169 to 175 °C, because it is not possible to sinter the material beyond this limit and the product cannot be made. Within these limits, the working chamber temperature did not affect the tensile properties (Figure 8 and Table 1). From the testing it can be concluded that the optimum temperature for the use of polyamides in the *Formiga P100* machine (of company *EOS* *GmbH Electro Optical Systems*, Krailling, Germany) is within the tested limits, so that all testing in this paper was done at a temperature of 172 °C.

The diagrams show only the average values of the test specimen of each series for better and easier comparison of the influence of processing parameters on the tensile properties.

**Figure 9:**
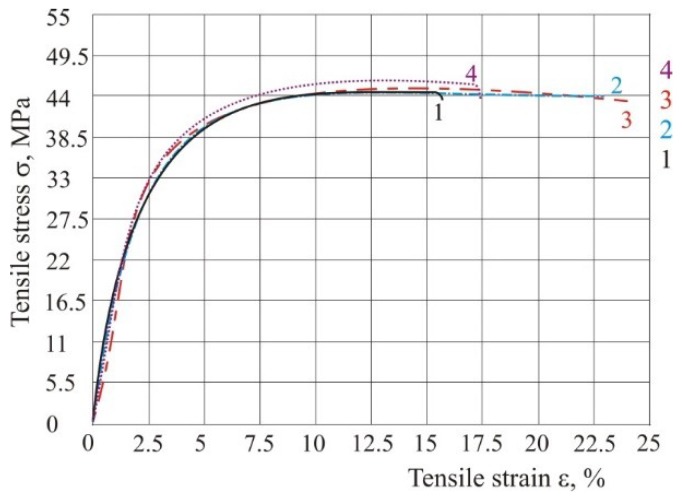
Tensile stress–strain curve with same energy according to Table 3.

If the parameters energy density and ratio of laser diameter and hatch distance do not change (Table 2), but the laser power and speed do change, then tensile properties remain the same (Table 3 and Figure 9). For this testing, hatch distance must be the same in all experiments, because if all three parameters change *ED* cannot be the same.

**Figure 10:**
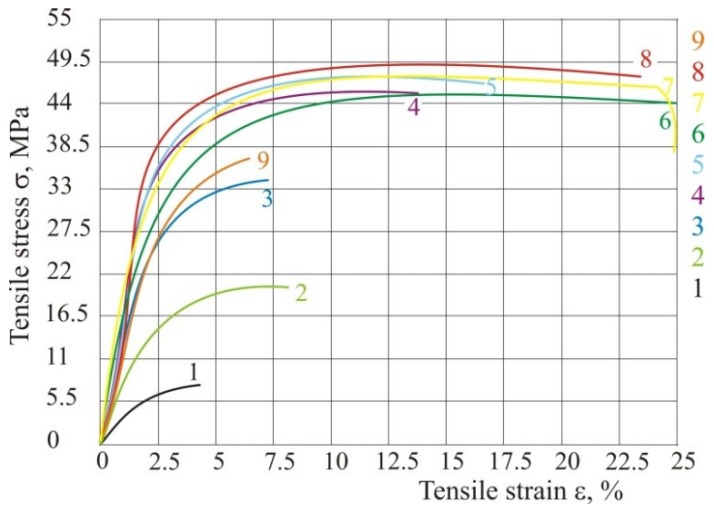
Tensile stress—strain curve with different energy according to Table 5.

If energy density has been changed (Table 4), the tensile properties are very different which can be seen in Table 5 and Figure 10, so we have confirmed the equation given by other authors from previous literature. For different energy density only one parameter can have a different value (e.g., laser power), all the others must be unchanged.

From Figure 10 it may be concluded that if energy is higher, then tensile properties are also higher. However, if we put excessive energy the tensile properties decrease (experiment 9)—tensile strain and stress decrease in comparison with test specimens 6, 7 and 8.

**Figure 11:**
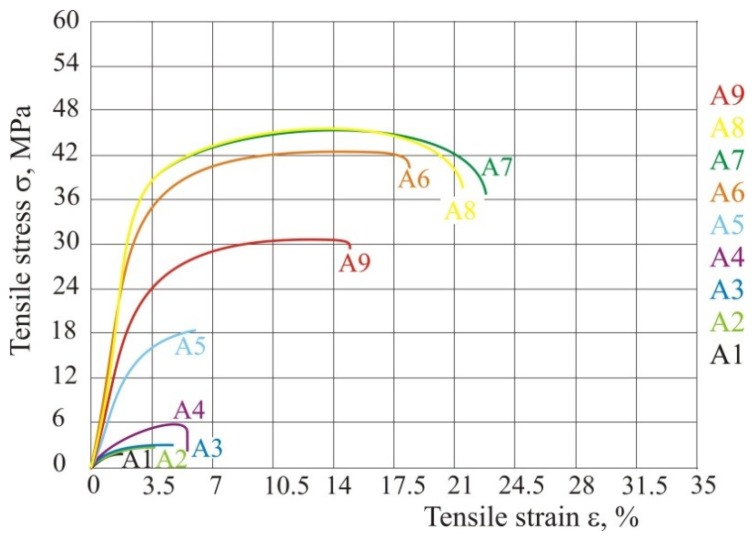
Tensile stress–strain curve with different beam overlay ratio according to Table 7.

To determine the influence of the overlay ratio and to test the new equation, the hatch distance is changed, and laser power and speed remain constant (Table 6). Because of constant laser beam diameter and the changing of the hatch distance, the energy density is also changed. Figure 11 shows the tensile properties when the beam overlay ratio is changed.

**Figure 12:**
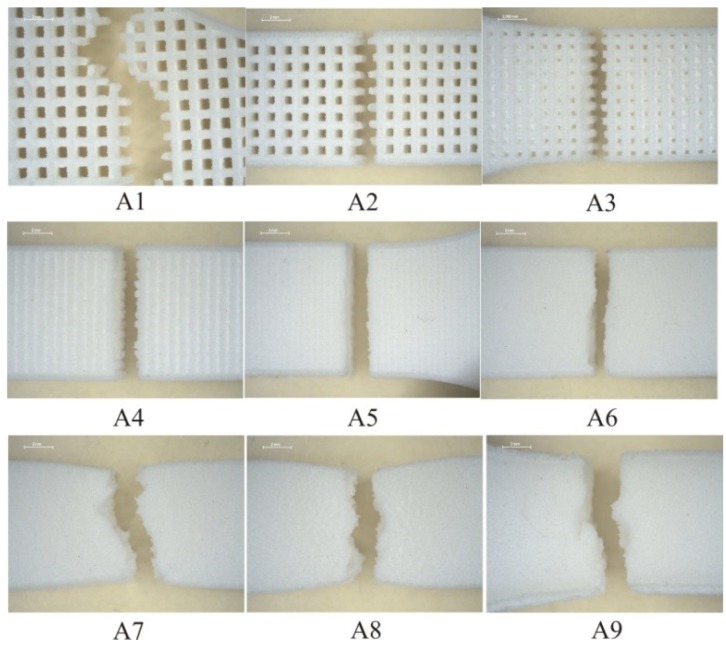
Microscopy images of fracture surface.

Microscopy images of fracture surface are shown in Figure 12. The images were made with device *Stereo Microscope Leica MZ6* with enlargement of 7.88×. For test specimens from experiment series A2 to A4 the fracture is a straight line within the chain made for hatch distance greater than laser beam diameter. In test specimen A5 we can see also a straight fracture because hatch distance is almost the same as the laser beam diameter, and the overlay ratio is near 1. After this value, the product will not have the mesh structure. All other tests (A6–A9) show partial arrangement of material macromolecular chains in the direction of force and narrowing occurs in the area of fracture.

**Figure 13:**
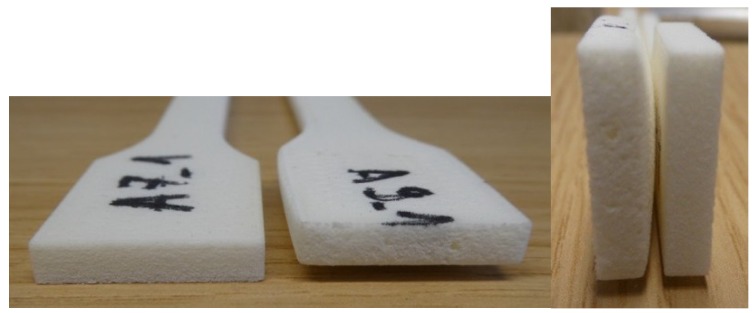
Geometrical deformation with higher energy input—comparison of one test specimen in series A7 and A9.

If we compare all these tables and figures the conclusion is that all parameters influence each other, and all parameters cannot be set, but some must be taken as a constant, because otherwise energy will not be the required number. Most important parameters are energy input that directly depends on the ratio of diameter and hatch distance. If overlay ratio is increased, tensile properties also increase. The best tensile properties are achieved between value of energy density of 0.0484 and 0.0667 J/mm^2^. If energy density is higher, tensile properties start to fall. This also influences the dimensions (test specimens 9 and A9) and of course the product mass. If there is excessive energy input the product does not only have bad tensile properties, but there are also significantly geometrical deformations (Figure 13). Figure 13 shows comparison of two products, left one with energy density 0.0484 J/mm^2^ and the right one with 0.1568 J/mm^2^. With higher energy input lots of deformation and dimensional deviations can be seen (Table 7).

To explain laser parameters a Differential Scanning Calorimetry (DSC) was carried out. Test specimens from series A4, A5, A8 and A9 were analyzed with the device *Mettler Toledo DSC 823e*. Every test sample with the mass of approx. 10 mg was heated with the heating rate of 10 °C/min in two cycles and then cooled down in a temperature range from 0 to 210 °C and N_2_ atmosphere.

**Figure 14:**
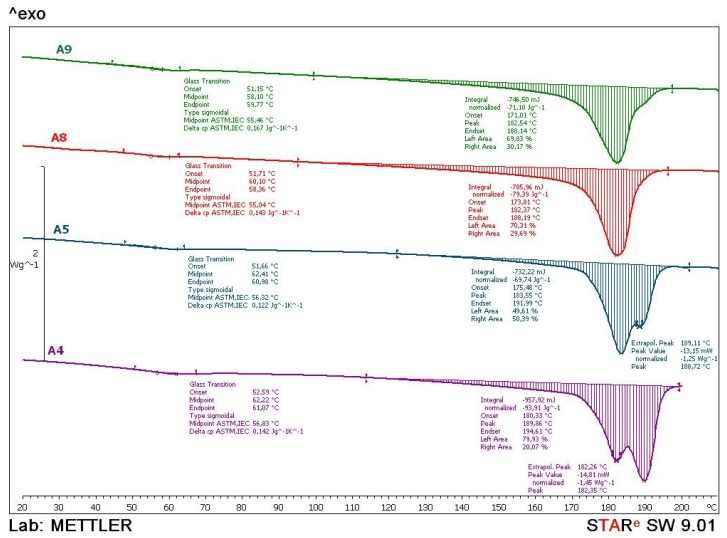
Comparative DSC thermograms of A4, A5, A8 and A9 samples obtained during the first heating cycle.

**Figure 15:**
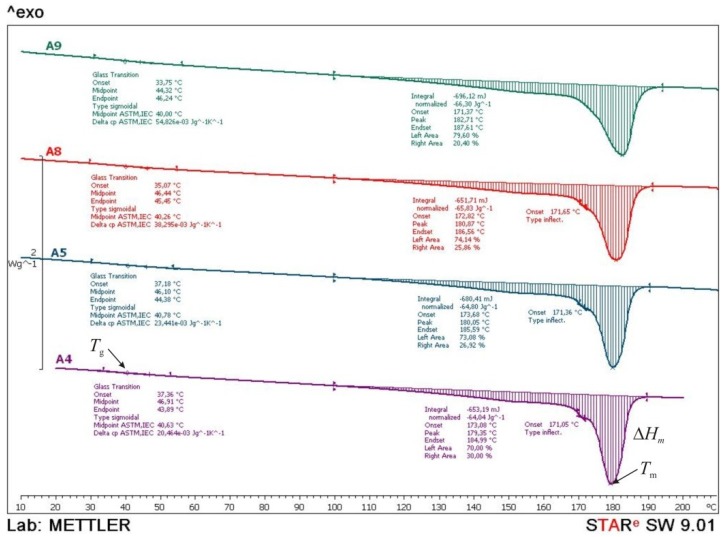
Comparative DSC thermograms of A4, A5, A8 and A9 samples obtained during the second heating cycle.

**Figure 16:**
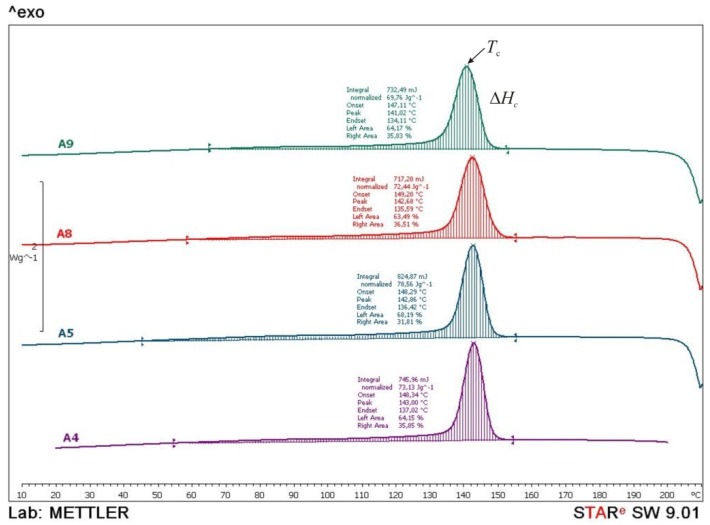
Comparative DSC thermograms of A4, A5, A8 and A9 samples obtained during the cooling cycle.

From the thermograms obtained in the first and second heating cycles the values of glass transition temperature (*T*_g_), melting temperature (*T*_m_) and melting enthalpy (Δ*H*_m_) were determined, while from the thermograms obtained in the cooling cycle crystallization temperature (*T*_c_) and associated crystallization enthalpy (Δ*H*_c_) were determined. The data obtained in the 2nd heating cycle are taken as relevant considering that the data obtained in the first heating cycle is to overcome the thermal history of the samples preparation. In Figure 14, Figure 15 and Figure 16, thermograms of the tested samples are shown and in Table 8 results of the *T*_g_, *T*_m_, Δ*H*_m,_ *T*_c_ and Δ*H*_c_ are given.

From the thermograms of the 1st heating cycle (Figure 14), it can be concluded that there are significant differences in the shape of the thermal transition which correspond to the melting of the crystalline phase of the polyamide, probably due to the different processing parameters of the samples. Glass transition temperatures are similar for all tested samples, approximately 56 °C. Compared to the values given by other authors for SLS of PA12 glass transition temperatures are similar to the ones obtained in this test [19,28,29]. In comparison with PA12 for classical processing, glass transition temperature is higher for about 20 °C. The higher value of the glass transition temperature results in the higher rigidity or lower flexibility of the macromolecules. The presence of the two maximums on the melting curve indicates the presence of different crystalline forms, of which the less arranged crystal forms are melted at the lower temperature while the more arranged crystals are melted at the higher temperatures. The values of melting enthalpy indicate the amount of crystal domains in polyamides. Higher values of melting enthalpy, i.e., a higher crystal structure, are found in the polyamide sample A4 which can be seen at right side of Figure 14.

After the 2nd heating cycle (Figure 15), the glass transition temperature of all samples lowers to the temperature of about 40 °C. The obtained results indicate an increase in the flexibility of polymeric (polyamide) macromolecules after the samples passed the first heating cycle. Values of melting temperature and melting enthalpy are similar for all tested samples, indicating the same polyamide structure.

During the cooling cycle (Figure 16), exothermic transition of crystallization occurs in a wide temperature range. The crystallization temperatures and crystallization enthalpy are similar in all tested samples (*T*_c_ = 141–143 °C and Δ*H*_c_ = 70–79 J/g). Similar crystallinity values, *T*_c_, indicate the same rate of crystallization. A little higher value of the crystallization enthalpy points to a slightly higher crystal phase in sample A5 (Δ*H*_c_ = 78.56 J/g) while lower values of crystallization enthalpy of other samples indicate a slightly lower crystal phase.

From the DSC results can be concluded that regardless the different processing parameters, all test specimens have equal thermal properties. Due to such equality in the results, the DSC analysis was not performed on other samples.

## 5. Discussion of the Results

Energy density *ED* is actually work *W* accomplished by power *P* in time *t* divided by scan spacing between each move of laser diameter and path of laser *ds*. This work *W* refers to the surface represented by the diameter of the laser beam and the total distance (path) passed by the laser, i.e., the area where laser sinters the product. Distance is speed multiplied by time, so that energy density is presented by the equation:

(4) $$\;ED=\frac{P\cdot t}{h\cdot ds}=\frac{P\cdot t}{h\cdot dv\cdot t}\;$$

where:

*ED*  energy density, J/mm^2^
*P*  laser power, W
*t*  time, s
*h*  scan spacing, hatch distance, mm
*ds*  path of the laser, mm
*dv*  laser beam speed, mm/s

However, this is only true if hatch distance *h* is equal to or greater than the laser beam diameter. In all other cases, the same path is run by the laser more than once or exactly in the ratio of the overlaying laser paths, which is shown in this paper as overlay ratio *x*, which is *x* = *d*/*h*, so proposed new equation for determination of energy density in SLS is:

(5) $$ED=\frac{P}{h\cdot dv}*\frac{d}{h}\;$$

In this equation, namely the laser speed is very important because of the viscosity of the liquid material (for example MFR), speed is not optional; it is within some limits. Because of that, energy density *ED* cannot be composed of any number (i.e., value of processing parameters—Table 2, Table 4 and Table 6).

Speed is also very important because the basis of additive manufacturing is the short production time. It is well known that if the laser speed is higher, the time of production will be shorter. However, it should be noted that production time depends not only on energy input calculated with laser power, speed, diameter of laser beam and hatch distance, but also on the size and volume of products in the chamber and of the product height in direction of the axis *z*. According to Figure 3, if hatch distance is higher, then the production time will be shorter, because laser sinters less space/material. However, higher hatch distance means lower tensile properties (Table 7 and Figure 11). Laser power is an implicit parameter and has no influence on the production time.

Laser diameter cannot be changed, so parameter hatch distance in overlay ratio is the important parameter. If hatch distance is smaller than laser diameter (Figure 3 and in Table 3 *h* < *d* (*h* = 0.23–0.33 mm)) then with overlay ratio between 0.03 to 0.7 J/mm^2^, the tensile properties will give maximum values. In Figure 3b in the first five test specimens one can see the geometric appearance if hatch distance is larger than laser diameter *h* > *d*. So, this has not only impact on the appearance but also on the mechanical properties that are incomparably small compared to *h* < *d* (e.g., tensile strength is only on average 3.5 MPa).

In the first heating cycle in DSC analysis the double peak indicates that there is far to less energy in part A4 and A5 because in this two parts there is a huge amount of non-molten particles. Those non-molten structures have a impact on mechanical properties.

**Figure 17:**
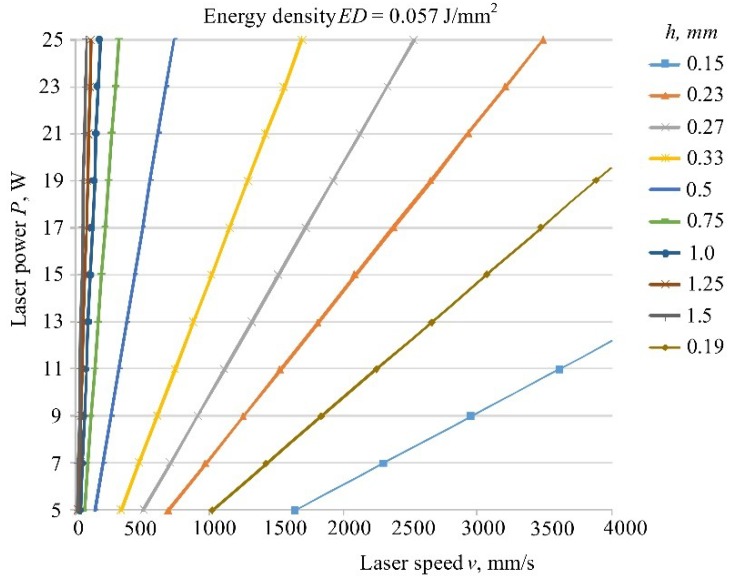
Selection of processing parameters in SLS.

Selections of the processing parameters cannot be taken as in the paper by authors Singh et al. because the deviation from the model will be very large. Because of that, Figure 17 shows the best choice for the selection of parameters (power, speed, and hatch distance) with constant laser beam diameter and energy density of 0.057 J/mm^2^. This value is the average value of two energy densities which gives the best tensile properties in the conducted experiment. The values of hatch distance were taken from Table 3. However, with this selection we must keep in mind what is the value of the laser beam diameters for the selected machine and in compliance with Figure 3 and of course with the production time.

This diagram is only one solution; higher values of speed and power can be added (so this can be used for laser with higher power). Furthermore, according to the presented equation other values/lines for the third parameter hatch distance can be added, not only one used in this experiment (for example in this picture hatch distance of 0.19 mm is added). In addition, all this can be done for some other energy densities.

## 6. Conclusions

Depending of the SLS working principle, apart from the parameters of power, speed and hatch distance, the properties are also affected by the laser beam diameter, so that the previous equation for the calculation of energy density must be expanded by the new factor, overlay ratio *x* which consists of diameter and hatch distance.

High tensile properties are achieved with high energy density. However, energy density should not exceed *ED* = 0.0667 J/mm^2^, since this results in material overheating, reduction of tensile properties and geometrical deformation.

The processing parameters: energy density, laser power, laser beam speed, hatch distance and layer thickness are determined according to the new mathematical equation and in practice the choice can be easily determined according to diagram in Figure 17.

Further papers should compare other manufacturing strategies apart from the manufacturing strategy *Sorted* and alternating direction of scanning the layer of polymer powder by the laser beam. Furthermore, it is necessary to see this equation with some other materials and blends that can be used in SLS, and the effect of other properties, not just mechanical.

## Figures and Tables

**Table 1:** Tensile properties with different chamber temperature.

No.	*h*, mm	*b*, mm	*A*_0_, mm^2^	*F*_m_, N	*R*_m_, MPa	*ε*_p_, %	*R*_p_, MPa	*E*, GPa
**175 °C**								
1	4.09	5.01	20.49	842.0	41.09	22.2	40.54	2.048
2	4.07	4.98	20.27	870.1	42.93	21.5	42.04	2.244
3	4.18	4.99	20.86	849.8	40.74	22.7	39.83	1.971
$\bar{x}$	4.11	4.99	20.54	853.97	41.59	22.13	40.80	2.088
*S*	0.059	0.015	0.298	14.506	1.176	0.603	1.128	0.141
**172 °C**								
1	4.03	5.10	20.55	873.7	42.09	22.8	41.55	2.053
2	4.10	5.06	20.75	880.4	42.44	22.7	41.35	2.012
3	4.06	5.09	20.67	868.0	42.00	22.9	40.48	2.034
$\bar{x}$	4.06	5.08	20.65	874.03	42.18	22.80	41.13	2.033
*S*	0.035	0.021	0.097	6.207	0.232	0.100	0.569	0.021
**169 °C**								
1	3.93	4.98	19.57	832.0	42.51	22.3	41.94	2.275
2	4.00	5.03	20.12	868.0	43.14	21.8	41.24	2.507
3	4.00	5.00	20.00	864.7	43.23	22.4	42.28	2.226
$\bar{x}$	3.98	5.00	19.90	854.9	42.96	22.17	41.82	2.336
*S*	0.040	0.025	0.288	19.901	0.392	0.321	0.530	0.150

**Table 2:** Processing parameters with the same value of energy density and overlay ratio.

No.	*P*, W	*v*, mm/s	*h*, mm	*x*	*ED*, J/mm^2^
1	15	2000	0.25	1.68	0.05
2	25	3333	0.25	1.68	0.05
3	7.5	1000	0.25	1.68	0.05
4	22.5	3000	0.25	1.68	0.05

**Table 3:** Tensile properties with the same value of energy density and overlay ratio.

No.	*h*, mm	*b*, mm	*A*_0_, mm^2^	*F*_m_, N	*R*_m_, MPa	*ε*_p_, %	*R*_p_, MPa	*E*, GPa
1_1	3.95	10.22	40.37	1817.0	45.01	15.04	42.32	1.601
1_2	3.95	10.26	40.53	1833.8	45.25	15.83	42.6	1.654
1_3	3.92	10.3	40.27	1835.5	45.46	16.5	42.7	1.647
$\bar{x}$	3.94	10.26	40.39	1828.8	45.24	15.79	42.54	1.634
*S*	0.017	0.040	0.128	10.247	0.225	0.731	0.197	0.029
2_1	3.96	10.2	40.39	1842.7	45.62	23.01	42.95	1.725
2_2	3.94	10.15	39.99	1819.6	45.5	23.35	43.17	1.751
2_3	3.96	10.25	40.79	1858.6	45.56	23.42	42.94	1.726
$\bar{x}$	3.96	10.2	40.39	1840.3	45.56	23.26	43.02	1.734
*S*	0.012	0.050	0.398	19.627	0.060	0.219	0.130	0.015
3_1	3.9	10.2	39.78	1817.5	45.69	24.3	43.02	1.641
3_2	3.92	10.16	39.83	1815.3	45.58	24	42.75	1.612
3_3	3.88	10.18	39.49	1806.7	45.74	24.48	43.17	1.616
$\bar{x}$	3.9	10.18	39.7	1813.2	45.67	24.26	42.98	1.623
*S*	0.020	0.020	0.181	5.714	0.082	0.242	0.213	0.016
4_1	3.9	10.22	39.86	1853.4	46.5	17.02	42.23	1.652
4_2	3.9	10.2	39.78	1846.6	46.42	16.78	42.65	1.629
4_3	3.96	10.3	40.48	1878.2	46.4	16.87	42.62	1.645
$\bar{x}$	3.92	10.24	40.04	1859.4	46.44	16.89	42.5	1.642
*S*	0.035	0.053	0.385	16.647	0.053	0.121	0.234	0.012

**Table 4:** Processing parameters with different energy.

No.	*P*, W	*v*, mm/s	*h*, mm	*x*	*ED*, J/mm^2^
1	7	3000	0.25	1.68	0.016
2	10.5	3000	0.25	1.68	0.024
3	14	3000	0.25	1.68	0.031
4	18	3000	0.25	1.68	0.040
5	21	3000	0.25	1.68	0.047
6	22	3000	0.25	1.68	0.049
7	22.5	3000	0.25	1.68	0.050
8	25	3000	0.25	1.68	0.056
9	22	1000	0.25	1.68	0.148

**Table 5:** Tensile properties with different energy.

No.	*h*, mm	*b*, mm	*A*_0_, mm^2^	*F*_m_, N	*R*_m_, MPa	*ε*_p_, %	*R*_p_, MPa	*E*, GPa
1_1	3.94	10.44	41.13	309.3	7.52	4.01	7.5	0.387
1_2	3.96	10.5	41.58	322.7	7.76	4.42	7.72	0.395
1_3	3.95	10.5	41.49	316.0	7.61	4.47	7.61	0.394
$\bar{x}$	3.95	10.48	41.4	316.0	7.63	4.3	7.61	0.392
*S*	0.010	0.035	0.235	6.668	0.121	0.252	0.110	0.004
2_1	3.92	10.1	39.59	805.7	20.35	8.5	20.32	0.953
2_2	3.92	10.2	39.98	824.5	20.62	8.62	20.6	0.938
2_3	3.95	10.12	39.97	819.3	20.50	8.56	20.37	0.944
$\bar{x}$	3.93	10.14	39.85	816.5	20.49	8.56	20.43	0.945
*S*	0.017	0.053	0.223	9.702	0.135	0.060	0.149	0.008
3_1	3.9	10.1	39.39	1340.0	34.02	7.24	34.0	1.321
3_2	3.9	10.06	39.23	1347.7	34.35	7.41	34.29	1.342
3_3	3.96	10.08	39.91	1374.6	34.44	7.31	34.34	1.336
$\bar{x}$	3.92	10.08	39.51	1354.1	34.27	7.32	34.21	1.333
*S*	0.035	0.020	0.352	18.130	0.221	0.085	0.184	0.011
4_1	3.94	10.14	39.95	1817.8	45.5	13.86	45.5	2.077
4_2	3.92	10.1	39.59	1806.6	45.63	13.24	45.58	2.025
4_3	3.93	10.03	39.41	1797.8	45.61	14.24	45.21	1.967
$\bar{x}$	3.93	10.09	39.65	1807.4	45.58	13.78	45.43	2.023
*S*	0.010	0.056	0.277	10.014	0.070	0.505	0.195	0.055
5_1	4.0	10.2	40.80	1927.8	47.25	16.68	47.2	1.811
5_2	3.98	10.2	40.60	1928.3	47.5	16.52	47.5	1.869
5_3	3.99	10.05	40.10	1906.3	47.54	16.96	44.5	1.825
$\bar{x}$	3.99	10.15	40.5	1920.8	47.43	16.72	46.4	1.835
*S*	0.010	0.087	0.358	12.569	0.157	0.223	1.652	0.030
6_1	3.98	10.2	40.60	1821.9	44.88	25.4	42.8	1.689
6_2	4.02	10.2	41.00	1855.4	45.25	25.5	44.23	1.715
6_3	4.0	10.26	41.04	1846.2	44.99	25.96	40.83	1.717
$\bar{x}$	4.0	10.22	40.88	1841.2	45.04	25.62	42.62	1.707
*S*	0.020	0.035	0.247	17.297	0.190	0.299	1.707	0.016
7_1	3.92	10.2	39.98	1848.9	46.24	24.01	39.68	1.748
7_2	3.9	10.2	39.78	1856.9	46.68	23.1	38.25	1.7
7_3	3.88	10.2	39.58	1848.4	46.7	25.22	33.1	1.808
$\bar{x}$	3.9	10.2	39.78	1851.4	46.54	24.11	37.01	1.752
*S*	0.020	0.000	0.204	4.795	0.260	1.065	3.461	0.054
8_1	4.0	10.16	40.64	1977.9	48.67	23.5	45.97	1.858
8_2	4.0	10.2	40.80	2009.4	49.25	23.58	46.56	1.888
8_3	4.0	10.18	40.72	1998.6	49.08	24.41	46.34	1.84
$\bar{x}$	4.0	10.18	40.72	1995.3	49.0	23.83	46.29	1.862
*S*	0.000	0.020	0.080	15.976	0.298	0.504	0.298	0.024
9_1	5.68	11.78	66.91	2401.4	35.89	6.5	35.89	1.211
9_2	5.7	11.5	65.55	2357.8	35.97	6.21	35.92	1.294
9_3	5.33	11.49	61.22	2232.3	36.44	6.94	36.34	1.35
$\bar{x}$	5.57	11.59	64.56	2330.5	36.1	6.55	36.05	1.285
*S*	0.208	0.165	2.972	87.831	0.297	0.368	0.252	0.070

**Table 6:** Conformation of new equation.

No.	*P*, W	*v*, mm/s	*h*, mm	*x*	*ED*, J/mm^2^
A1	21	2500	1.50	0.28	0.0016
A2	21	2500	1.25	0.34	0.0023
A3	21	2500	1.00	0.42	0.0035
A4	21	2500	0.75	0.56	0.0063
A5	21	2500	0.50	0.84	0.0141
A6	21	2500	0.33	1.27	0.0324
A7	21	2500	0.27	1.56	0.0484
A8	21	2500	0.23	1.83	0.0667
A9	21	2500	0.15	2.80	0.1568

**Table 7:** Tensile properties with new equation.

No.	*h*, mm	*b*, mm	*A*_0_, mm^2^	*F*_m_, N	*R*_m_, MPa	*ε*_p_, %	*R*_p_, MPa	*E*, GPa
A1_1	4.15	9.92	41.17	90.6	2.2	1.47	1.87	-
A1_2	4.18	9.94	41.55	101.0	2.43	1.65	1.35	-
A1_3	4.18	9.9	41.39	85.1	2.06	1.68	1.43	-
$\bar{x}$	4.17	9.92	41.37	92.2	2.23	1.6	1.55	-
*S*	0.017	0.020	0.192	8.074	0.187	0.114	0.280	-
A2_1	4.2	9.9	41.58	124.3	2.99	3.41	2.98	-
A2_2	4.24	9.86	41.81	131.3	3.14	3.6	3.14	-
A2_3	4.22	9.88	41.68	129.0	3.11	3.73	2.94	-
$\bar{x}$	4.22	9.88	41.69	128.2	3.08	3.58	3.02	-
*S*	0.020	0.020	0.113	3.543	0.079	0.161	0.106	-
A3_1	4.2	9.9	41.58	141.8	3.41	4.52	3.25	-
A3_2	4.26	9.86	42.00	147.9	3.52	4.68	3.48	-
A3_3	4.44	9.85	43.74	155.9	3.57	4.75	3.44	-
$\bar{x}$	4.3	9.87	42.44	148.5	3.5	4.65	3.39	-
*S*	0.125	0.026	1.143	7.058	0.082	0.118	0.123	-
A4_1	4.32	9.86	42.60	215.1	5.05	5.29	2.04	0.315
A4_2	4.36	9.88	43.08	223.6	5.19	5.3	1.84	0.298
A4_3	4.4	9.72	42.79	222.5	5.21	5.79	1.49	0.308
$\bar{x}$	4.36	9.82	42.82	220.4	5.15	5.46	1.79	0.307
*S*	0.040	0.087	0.242	4.615	0.087	0.286	0.278	0.009
A5_1	4.06	9.9	40.19	744.0	18.51	5.88	18.42	0.8
A5_2	4.08	9.86	40.23	754.3	18.75	5.96	18.69	0.837
A5_3	4.07	9.79	39.85	748.4	18.78	5.92	18.66	0.838
$\bar{x}$	4.07	9.85	40.09	748.9	18.68	5.92	18.59	0.825
*S*	0.010	0.056	0.211	5.166	0.148	0.040	0.148	0.022
A6_1	4.1	9.9	40.59	1744.6	42.98	18.25	41.01	1.511
A6_2	4.06	9.92	40.28	1747.9	43.4	18.52	40.63	1.426
A6_3	4.08	9.91	40.42	1735.6	42.92	18.31	40.67	1.518
$\bar{x}$	4.08	9.91	40.43	1742.7	43.1	18.36	40.77	1.485
*S*	0.020	0.010	0.157	6.379	0.262	0.142	0.209	0.051
A7_1	4.2	10.0	42.00	1912.7	45.54	22.8	36.85	1.6
A7_2	4.16	10.02	41.68	1918.7	46.03	23.02	36.98	1.587
A7_3	4.03	9.98	40.22	1849.4	45.98	22.61	36.09	1.598
$\bar{x}$	4.13	10.0	41.3	1893.6	45.85	22.81	36.64	1.595
*S*	0.089	0.020	0.951	38.359	0.270	0.205	0.481	0.007
A8_1	4.2	10.1	42.42	1950.9	45.99	21.5	37.85	1.798
A8_2	4.2	10.12	42.50	1930.1	45.41	21.36	37.2	1.821
A8_3	4.23	10.17	43.03	1948.0	45.28	21.52	37.03	1.784
$\bar{x}$	4.21	10.13	42.65	1943.0	45.56	21.46	37.36	1.801
*S*	0.017	0.036	0.328	11.260	0.378	0.087	0.433	0.019
A9_1	4.9	10.9	53.41	1669.1	31.25	14.77	30.1	1.099
A9_2	4.92	10.92	53.73	1656.4	30.83	14.25	29.82	1.115
A9_3	4.94	10.88	53.75	1663.6	30.95	15.56	29.84	1.038
$\bar{x}$	4.92	10.9	53.63	1663.0	31.01	14.86	29.92	1.084
*S*	0.020	0.020	0.191	6.357	0.216	0.660	0.156	0.041

**Table 8:** Results of DSC measurements.

No.	1. Heating Cycle (Figure 14)			2. Heating Cycle (Figure 15)			Cooling Cycle (Figure 16)	
	*T*_g_ (°C)	*T*_m_ (°C)	*ΔH*_m_ (J/g)	*T*_g_ (°C)	*T*_m_ (°C)	*ΔH*_m_ (J/g)	*T*_c_ (°C)	*ΔH*_c_ (J/g)
A4	56.8	182.4 189.9	93.9	40.6	179.4 171.1	64.0	143.0	73.1
A5	56.3	183.6 188.7	69.7	40.8	180.1 171.4	64.8	142.9	78.6
A8	55.0	182.4	79.4	40.3	180.9 171.7	65.8	142.7	72.4
A9	55.5	182.5	71.1	40.0	182.7 171.0	66.3	141.0	69.8
